# Thermal Properties and Features of Nanofluids

**DOI:** 10.3390/nano15050334

**Published:** 2025-02-21

**Authors:** S M Sohel Murshed

**Affiliations:** IDMEC, Instituto Superior Técnico, University of Lisbon, 1049-001 Lisbon, Portugal; smurshed@tecnico.ulisboa.pt

Nanofluids have emerged as an advanced media in many applications, particularly thermal management and energy efficiency applications, with extensive research focusing on their thermophysical properties and thermal performances. The collection of studies presented in this Special Issue explores various aspects of nanofluids, from hybrid nanoparticle compositions to their stability, heat transfer enhancement, phase change features and recyclability. These advancements hold the potential to revolutionize existing industrial applications, particularly in fields that require highly efficient thermal management and energy conversion technologies.

Despite being introduced nearly three decades ago, nanofluids remain a dynamic and evolving field of research. Although extensive studies have been conducted, real-world applications are yet to be fully realized due to significant challenges, including stability issues, inconsistencies in results, inconclusive underlaying mechanisms and concerns regarding their compatibility with conventional and micro and mesoscale systems [1]. Most investigations have demonstrated notable improvements in thermophysical properties, but the presence of scattered and inconsistent data prevents the establishment of definitive conclusions regarding the mechanisms driving these enhancements. The primary hurdle remains the development of nanofluids with long-term stability and consistent performance over extended periods. Addressing these challenges is crucial for unlocking their full potential in numerous practical applications. Beyond their thermophysical properties, their thermal transport features such as convection and boiling have exhibited considerable improvements across reported studies [2]. These attributes are fundamental to positioning nanofluids as the next generation of cooling agents.

The objective of this Special Issue was to provide a comprehensive overview of nanofluids, emphasizing their critical thermal properties, key challenges and diverse applications. By covering a broad spectrum of topics, this Special Issue serves as a valuable resource for researchers and professionals seeking to deepen their understanding of nanofluid technology and its prospects.

The first study on thermal conduction in hybrid nanofluids and aggregates investigates the role of particle aggregation in optimizing thermal conductivity [3]. By employing a meshless computational method and core–shell cluster formations, the research reveals how the spatial distribution and fractal dimension of aggregates significantly influence the overall heat conduction performance of hybrid nanofluids. This work underscores the importance of nanoparticle dispersion conditions in achieving superior thermal efficiencies. The findings suggest that controlled aggregation methods may allow for the design of nanofluids with tailored conductivity profiles, thus enhancing their performance in various engineering applications, and more specifically in thermal management.

Expanding on functionalized nanofluids, the study in contribution [4] on TiO_2_ nanoparticles in silylalkyl bridged polyaniline-based nanofluids introduces an innovative nanocomposite that enhances the stability, thermal and optical properties of the nanofluid. By incorporating polyaniline-modified TiO_2_ nanoparticles, the research highlights improved dispersion, controlled aggregation and synergistic enhancements in the nanofluids’ thermal conductivity, refractive index and rheological behavior. These findings suggest promising applications in advanced heat transfer and electronic cooling systems, potentially leading to next-generation thermal management solutions in electronics and industrial cooling processes.

In the context of flow boiling heat transfer, the experimental investigation carried out in contribution [5] explores the use of nanorefrigerants based on n-pentane and various nanomaterials and it demonstrates significant improvements in flow boiling heat transfer performance. The transition from forced convection to nucleate boiling, driven by nanoparticle interactions with the heater surface, marks a notable advancement in the application of nanorefrigerants for efficient phase-change-based cooling technologies. The study provides important insights into the mechanisms governing heat transfer enhancement, paving the way for more effective and sustainable cooling strategies in energy-intensive applications.

The study on the stability and thermophysical properties of a GNP-Fe_2_O_3_ hybrid nanofluid further advances our understanding of hybrid nanofluids by examining the effects of concentration and temperature on electrical conductivity, viscosity and thermal conductivity [6]. The results highlight the superior thermophysical properties of these fluids, reinforcing their potential for industrial applications requiring efficient heat transfer techniques. The study highlights the tunability of hybrid nanofluids, demonstrating their ability to enhance performance across a range of operating conditions, making them suitable for diverse engineering applications such as heat exchangers and cooling systems.

Finally, the investigation into pool boiling heat transfer of new and recycled Alumina nanofluids introduces an environmentally conscious approach by assessing the recyclability of this nanofluid [7]. The study demonstrates substantial enhancements in boiling critical heat flux while highlighting the feasibility of reusing nanofluids without a significant reduction in their performance. This work paves the way for the sustainable application of nanofluids in heat transfer systems, addressing the growing need for eco-friendly and cost-effective solutions to industrial thermal management with advanced media like nanofluids. The potential to reuse nanofluids without major performance degradation presents a compelling case for their adoption in long-term operational scenarios.

Collectively, these studies contribute valuable insights into the development and application of nanofluids, offering new perspectives on their role in enhancing the thermal and functional properties of materials across multiple application fields. The innovations presented here hold great promise for future advancements in thermal management, energy-efficient systems and sustainable nanotechnology solutions. By leveraging these cutting-edge findings, industries can harness the full potential of nanofluids to optimize thermal management and improve overall energy conversion efficiency.
